# Proportions of CD4+, CD8+ and B cell subsets are not affected by exposure to HIV or to Cotrimoxazole prophylaxis in Malawian HIV-uninfected but exposed children

**DOI:** 10.1186/s12865-015-0115-y

**Published:** 2015-08-28

**Authors:** Herbert Longwe, Kamija S. Phiri, Nyanyiwe M. Mbeye, Thandile Gondwe, Kondwani C. Jambo, Wilson L. Mandala

**Affiliations:** Department of Basic Medical Sciences, College of Medicine, University of Malawi, Blantyre, Malawi; Tropical Haematology Research Unit, College of Medicine, University of Malawi, Blantyre, Malawi; Department of Public Health, College of Medicine, University of Malawi, Blantyre, Malawi; Malawi-Liverpool-Wellcome Trust Clinical Research Programme, Blantyre, Malawi

## Abstract

**Background:**

As a result of successful PMTCT programs, children born from HIV-infected mothers are now effectively protected from contracting the infection. However, it is not well known whether *in utero* exposure to the virus and the subsequent exposure to Cotrimoxazole (CTX) prophylaxis affect the cell mediated immune system of the children. This observational prospective study was aimed at determining how CD4^+^ T, CD8^+^ T and B cell subsets varied in HIV-exposed but uninfected (HEU) children at different ages.

**Methods:**

We recruited HEU and HIV-unexposed and uninfected (HUU) children from 6 months of age and followed them up until they were 18 months old. HEU children received daily CTX prophylaxis beginning at 6 weeks of age until when 12 months of age. Venous blood samples were collected 6 monthly and analysed for different subsets of CD8^+^ T, B cells and totalCD4^+^ T cells.

**Results:**

At 6 months of age, HEU children had a lower percentage of total CD4^+^ T cells compared to HUU children and a lower proportion of naïve CD8^+^ T cells but higher percentage of effector memory CD8^+^ T cells compared to HUU children. HEU and HUU children had similar proportions of all B cell subsets at all ages.

**Conclusions:**

The study showed that the subtle variations in CD4^+^ and CD8^+^ T cell subsets observed at 6 months do not last beyond 12 months of age, suggesting that HEU children have a robust cell-mediated immune system during first year of life.

**Trial registration:**

This article report is not based on results of a controlled health-care intervention

## Background

Lately the number of HIV-exposed but uninfected (HEU) children (HIV negative children born from HIV-infected mothers) has substantially increased due to the scaling up of prevention of mother to child transmission (PMTCT) programs [1]. Although HEU children are not infected with the virus, some studies have still shown that the immune system of these children might have long-term changes either due to *in utero* exposure to HIV viral particles or antiretroviral (ART) exposure [2]. These changes are thought to have a negative effect on the response to infections as evidenced by the high morbidity in HEU children early in life [3, 4]. However, most of these studies were predominantly undertaken in HEU neonates and during the era before the advent of daily Cotrimoxazole (CTX) prophylaxis for HEU children and that of lifelong ART for pregnant women and lactating mothers.

The adaptive immune system’s success in combating infection relies heavily on putting up a robust response and timely and appropriate differentiation of the B and T lymphocytes into specific phenotypes. T helper (CD4^+^) and T cytotoxic (CD8^+^) T cells are classified either as naïve, effector or memory based on the immune function they perform [5]. Naïve T cells, characterized by the expression of CD45RA^+^, continuously circulate between the secondary lymphoid organs and blood via the lymphatic system [6] and recognize antigens during infection [7]. The naïve T cells are then activated, expand and differentiate into either short-lived effector T cells or memory precursor effector T cells that survive for a longer period and provide protection during re-infection with the same pathogen [8]. Memory T cells are classified either as effector memory (T_EM_) (CD45RA^−^CCR7^−^) or central memory (T_CM_) T cells (CD45RA^−^CCR7^+^) [9].

Depending on age, circulating human B cells in healthy individuals comprise naïve (CD19^+^CD27^−^CD21^hi^CD10^−^), and memory B cells that express either unswitched or switched antibody isotypes [10–12]. A population of plasma cells that represent the terminally differentiated mature effector B cells and express CD19^+^CD27^+^CD38^hi^CD10^−^ is found in very low numbers [10, 13]. Protective humoral immune response with optimal longevity depends on generation of memory B cells that are classified into classical memory that are typically CD19^+^CD27^+^CD21^hi^CD10^−^ and activated memory expressing CD19^+^CD27^+^CD21^lo^CD10^−^ [14]. Transitional immature B cells, a minor population of circulating B cells, express an immature phenotype (CD19^+^CD27^−^CD21^lo^CD10^+^) and have reduced capacity to be activated [10]. Recently a unique memory B cell sub-population characterized by surface markers CD19^+^CD27^−^CD21^lo^CD10^−^ and defined by the expression of the inhibitory receptor Fc-receptor-like-4 (FCRL4) has been identified [15].

Currently little is known about the phenotypic changes of B and T cell populations in peripheral blood of HEU children in the era of lifelong ART in pregnancy and lactating mothers and also in HEU children on CTX prophylaxis during the first year of life. Although CTX has been shown to prevent opportunistic bacterial infections in HIV-infected individuals [16–18] little is known of its effect on the development of the immune system in HEU children. Therefore, the aim of this study was to characterize B and T cell phenotypic changes in HEU Malawian children during and after completion of daily Cotrimoxazole prophylaxis.

## Methods

### Study site, participants and design

Details of the study site and participants have already been reported previously [19]. Briefly, we recruited HEU children and HUU children from 6 months of age and followed them up until they were 18 months old in Zomba, Malawi. HEU children who had confirmed PCR negative results were randomly selected from the ART clinic at the Zomba central hospital. The children received daily CTX prophylaxis beginning at 6 weeks of age until at 12 months of age where we conducted a rapid test to rule out HIV infection and the mothers were asked to stop breastfeeding. The children were eligible for inclusion if there were otherwise healthy, breastfeeding, not under any medication and if the mothers indicated their intention to reside in the catchment area for the duration of the study. HUU children were those that were HIV-uninfected and recruited from HIV-uninfected mothers residing in the same area as HEU children. Those who were severe malnourished, anaemic, or who presented with other illnesses such as malaria, diarrhea or influenza were excluded from the study. In addition, only those who had not received any malaria medication during the two months before recruitment were included in the HUU group. Both HEU and HUU children were seen every six months and at each visit, 1 ml of venous blood was collected in EDTA tubes for immunophenotyping to characterize the T and B cell subsets.

### Ethical approval

The study was reviewed and approved by the College of Medicine Research Ethics Committee (COMREC) (P.05/10/954). Individual written informed consent was obtained from the parents or guardians of all the children who participated in the study.

### T and B cell characterisation

Whole blood samples were labelled with anti CD3 PE, anti CD4 PerCP, anti CD8 APC-H7 (all from BD Biosciences, San Jose, California), anti CD45RA FITC (BD Pharmingen, San Jose, California) and anti CCR7 APC (eBiosciences, San Diego, California). The following classification was used for different cell types; T helper (CD3^+^CD4^+^) and T cytotoxic (CD3^+^CD8^+^). The following additional criteria was used for the different subsets; naïve (CD45RA^+^CCR7^+^), central memory (CD45RA^−^CCR7^+^), effector memory (CD45RA^−^CCR7^−^) and terminally differentiated effector cells (CD45RA^+^CCR7^−^).

For the B cells, whole blood was labelled with anti CD19 APC, anti CD21 PE-cy5 (all from BD Pharmingen, San Jose, California), anti CD10 FITC and anti CD27 PE (eBiosciences, San Diego, California). B cells were identified as CD19^+^ and subpopulations of naïve as CD19^+^CD27^−^CD21^hi^CD10^−^, classical memory as CD19^+^CD27^+^CD21^hi^CD10^−^, activated memory as CD19^+^CD27^+^CD21^lo^CD10^−^, atypical memory CD19^+^CD27^−^CD21^lo^CD10^−^ and immature transitional as CD19^+^CD27^−^CD21^lo^CD10^+^.

### Statistical analysis

Statistical analysis and graphical presentation were done using GraphPad Prism 6 (GraphPad, California, USA) and STATA (StataCorp, Texas, USA). Mann-Whitney *U* test was used for comparison between the study groups at each visit. Results are presented as medians and interquartile ranges. Any differences between the study groups were considered significant if *p* values were less than 0.05.

## Results

**Fig. 1:**
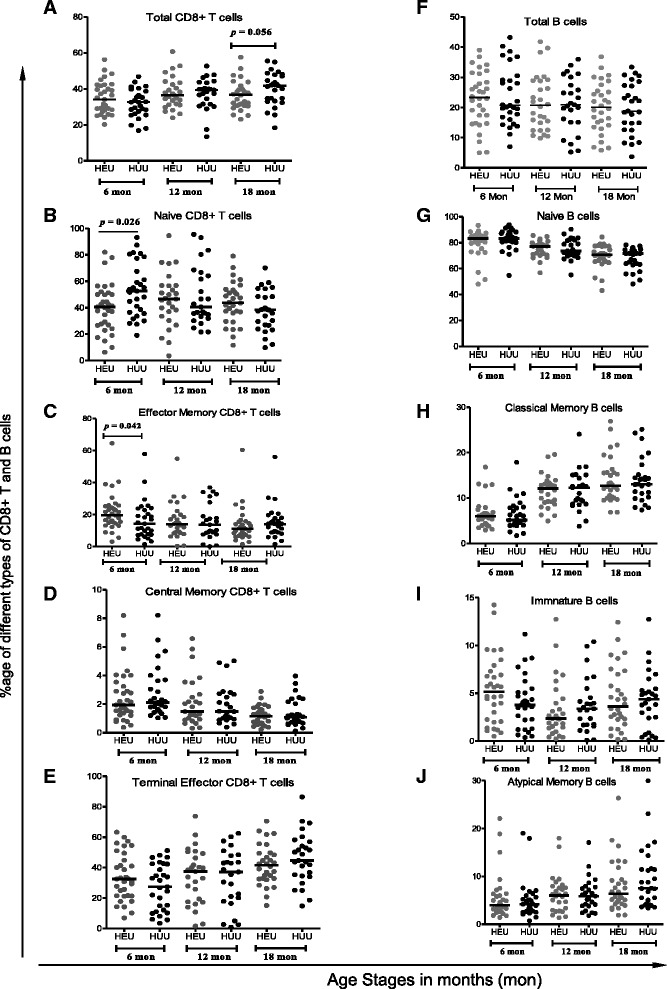
Proportions of different subsets of CD8^+^ T cells and B cells in HEU and HUU children at different age: Figures **a** represents percentage of CD8^+^T cells out of total lymphocytes, **b** naïve CD8^+^ T cells, **c** effector memory CD8^+^ T cells, **d** central memory CD8^+^ T cells and **e** terminal effector CD8^+^ T cells. Figures **f** represents proportion of CD19^+^ B cells as a percentage of total lymphocytes, **g** naive B cells, **h** classical memory B cell, **i** immature transitional B cells and **j** atypical memory B cells. Horizontal bars represent medians. Comparison between groups was performed with Mann Whitney *U* test. A difference with a *p* value of less than 0.05 was considered to be statistically significant

Demographic details of the study participants have already been reported previously [19]. At 6 months of age, the median proportion of the total CD4^+^ T cells in HEU was significantly lower compared to HUU children (55.2 vs. 58.4 %, *p* = 0.04) (Table 1). We did not find any significant differences in the CD4 T cell subsets between the two groups at all time points (Data not shown). Median proportion of CD8^+^ T cells was similar between HEU children compared to their HUU counterparts (35.1 vs. 32.6 %, *p* = 0.15) (Fig. 1a). No differences were observed in the median proportions of total CD4^+^ and CD8^+^ T cells between HEU and HUU children at 12 months: CD4^+^ (54.8 vs. 51.1 %, *p* = 0.621), CD8^+^ (36.4 vs. 39.3 %, *p* = 0.634) and 18 months:CD4^+^(54.8 vs. 48.8 %, *p* = 0.052), CD8^+^ T cells (36.7 vs. 41.7 %, *p* = 0.056) (Table 1). HEU children had significantly higher ratio of CD4:CD8 (*p* = 0.05) compared to HUU children (Table 1).

**Table 1:** Comparison of the medians (Interquartile Range) of various cell type subsets at different time points

Cell Type or Ratio		6 Months			12 Months			18 Months		
		HEU	HUU	*p*	HEU	HUU	*p*	HEU	HUU	*p*
Total CD4+ T cells		55.20 (47.50 - 62.80)	58.40 (55.20 – 67.50)	**0.04**	54.80 (42.70 – 59.30)	51.10 (44.60 – 54.90)	0.62	54.80 (47.70 – 58.70)	48.80 (39.50 – 55.30)	**0.05**
Effector memory CD4+ T cells		6.8 (5.13 – 9.29)	6.47 (4.38 – 8.93)	0.53	9.7 (6.01 – 13.2)	7.64 (5.30 – 10.2)	0.19	11.9 (10.2 – 14.7)	12.1 (9.24 – 16.5)	0.89
CD4:CD8 Ratio		1.57 (1.16 – 2.18)	1.79 (1.47 – 2.54)	0.08	1.50 (1.00 – 1.90)	1.30 (1.05 – 1.78)	0.64	1.51 (1.10 – 1.92)	1.18 (0.80 - 1.63)	**0.05**
CD8+ T cells Subsets	Total CD8+ T	35.10 (29.0 – 42.3)	32.60 (27.40 – 36.40)	0.15	36.40 (31.20 – 43.40)	39.30 (31.50 – 43.40)	0.63	36.70 (30.60 – 39.90)	41.70 (33.20 – 47.90)	**0.05**
	Naïve	41.40 (27.6 – 51.3)	52.60 (36.50 – 67.50)	**0.02**	46.60 (33.50 – 56.80)	40.50 (33.40 – 64.20)	0.88	43.80 (36.00 – 53.30)	38.40 (26.70 – 48.50)	0.17
	Central memory	1.94 (1.37 – 3.24)	2.11 (1.80 – 3.63)	0.38	1.48 (0.94 – 2.68)	1.48 (1.01 – 2.68)	0.82	1.17 (0.67 – 1.62)	1.08 (0.74 – 2.16)	0.75
	Effector memory	19.50 (14.90 – 25.30)	14.30 (7.65 – 22.8)	**0.04**	14.10 (8.51 – 20.0)	13.70 (8.68 – 19.0)	0.89	11.20 (7.37 – 15.6)	14.10 (8.85 – 18.0)	0.28
	Terminal Effector	32.50 (21.50 – 44.80)	27.40 (12.1 – 42.3)	0.15	37.40 (17.50 – 49.30)	37.20 (20.20 – 47.80)	0.96	41.60 (32.60 – 49.40)	44.70 (34.50 – 57.00)	0.29
B cells Subsets	Total CD19+ B cells	23.30 (16.10 – 29.80)	20.7 (16.8 – 29.10)	0.91	20.70 (13.70 – 29.10)	20.90 (15.20 – 27.40)	0.98	20.10 (14.10 – 25.50)	18.80 (12.60 – 27.30)	0.91
	Naïve	83.10 (79.20 – 85.60)	83.16 (80.70 – 87.90)	0.35	77.15 (71.20 – 78.90)	73.55 (70.60 – 81.90)	0.98	70.75 (66.40 – 77.20)	71.53 (63.20 – 74.00)	0.45
	Immature/Transitional	5.14 (2.14 – 7.11)	3.78 (2.21 – 5.09)	0.12	2.35 (1.39 – 4.19)	3.38 (1.70 – 4.54)	0.32	3.63 (2.26 – 6.98)	4.39 (2.41 – 5.47)	0.91
	Classical Memory	5.98 (5.29 – 6.73)	5.26 (4.59 – 7.85)	0.58	12.11 (9.09 – 13.30)	12.27 (8.68 – 14.4)	0.92	12.72 (10.2 – 15.6)	13.05 (10.20 – 15.20)	0.95
	Activated Memory	1.32 (0.77 – 2.41)	1.37 (0.85 – 2.16)	0.98	3.21 (2.12 – 3.85)	2.77 (1.64 – 3.78)	0.36	3.17 (2.42 – 4.53)	4.00 (3.17 – 5.22)	0.12
	Atypical Memory	3.96 (2.65 – 6.17)	4.21 (2.75 – 5.65)	0.91	5.99 (2.86 – 7.78)	5.86 (3.84 – 7.77)	0.91	6.38 (4.28 – 9.34)	7.53 (4.28 – 11.6)	0.34

The *p* values presented in bold indicate statistically significant differences between the HEU and HUU values for each cell type

At 6 months, there was significantly lower proportion of naïve CD8^+^T cells (41.4 vs. 52.6 %, *p* = 0.02) in HEU children compared to HUU children (Fig. 1b). Interestingly, a significantly high median proportion of T_EM_ CD8^+^T cells (Fig. 1c) was observed in HEU children at 6 months compared to HUU children (19.5 vs. 14.3 %, *p* = 0.04). No significant differences were observed between HEU and HUU children in median proportions of the remaining CD8^+^ T subsets (Fig. 1b–e) at 12 and 18 months of age or effector memory (T_EM_) CD4+ T cells (Table 1), either at 6, 12 or 18 months stages.

Median proportions of CD19^+^ B cells were similar between HEU children compared to their HUU counterparts at 6 months (23.3 vs. 20.7 %, *p* = 0.91), 12 months (20.7 vs. 20.9 %, *p* = 0.98) and 18 months (20.1 vs. 18.8 %, *p* = 0.91). No significant differences were observed in the median proportions of all B cell subsets (Fig. 1g–j), in HEU children compared with HUU children at all three time points.

## Discussion

HEU children have been reported to present with immune abnormalities during infancy, which are thought to be associated with *in utero* exposure to HIV. We investigated how B and T cell phenotypes from peripheral blood of HEU children vary during and after CTX prophylaxis in comparison with proportions in HUU children. We found that the overall distribution of B and T cell phenotypes was similar among HEU and HUU children at all time points with some few exceptions. The proportion of total CD4^+^ T cells was significantly lower in HEU children at 6 months stage. HEU children had a lower proportion of naïve CD8^+^ T cells at six months which steadily increased with age. Of much interest was the finding that HEU children had higher proportion of effector memory CD8^+^ T cells compared to HUU children at 6 months of age.

One study found that HEU Mozambican infants had reduced percentage of CD4^+^ T cells at 1 month compared to HUU children [20] and another study showed that total CD4^+^ T cell counts were lower in HEU children in early infancy compared to HUU children [21]. Exposure to maternally derived HIV proteins during pregnancy has been suggested to be one possible cause of the reduction in CD4^+^ T cell counts in HEU children [21–23]. Our findings support the idea that HEU children may have lower proportions of CD4^+^ T cells during early infancy [24, 25], but this abnormality seems not to last for longer than a year.

We also found that the proportions of naïve CD8^+^ T cells were significantly reduced, whereas that of effector memory CD8^+^ T cells was significantly higher, in HEU children compared to HUU at 6 months of age. These results support the hypothesis that there may be exposure of the foetal immune system to HIV viral proteins *in utero* and this may lead to immune activation that drives CD8^+^ T cells into expansion and differentiation into effector cells [26–28]. However, we expected the *in vitro* exposure to have equally, if not in a more significantly manner, affected the proportion of effector memory CD4+ T cells too. The fact that we did not observe this might be explained by the fact that these HIV-infected mothers were on ART during their pregnancy and during the time they were breastfeeding the HEU children.

Alterations in the percentage of B lymphocytes in HIV exposed neonates have been reported before [29] in a study that showed B cells increased in cord blood of HIV exposed neonates, supposedly caused by an increase in CD19^+^CD5^+^ B cells. Higher CD19^+^ B cell percentages among HEU infants (2 to 6 months) born to mothers with high viral load (>1000 copies/ml) at the time of delivery have also been reported [26]. The current study showed no evidence of CD19^+^ B cell subset alteration in HEU children at 6, 12 and 18 months of age. This may imply that the B cells ability to produce antibodies may not be impaired in HEU children.

The study had a number of limitations. Firstly, no data on lymphocyte subset proportions at birth for the children were collected, as this would have provided an ideal baseline for comparison with the subsequent follow-up stages. Secondly, although all the HIV-infected mothers were confirmed to be on ART, no data on CD4 T cell counts or HIV viral load levels, or duration on ART were collected from the mothers at the time of giving birth and during the follow up period of the study. Maternal disease status, especially their viral load at the time of recruitment, might have affected the extent of infant HIV exposure *in utero* and through breast milk [26]. As such it is not easy for the study to ascertain whether the lack of differences in the B and T cell phenotypes observed in the HEU group was due to reduced exposure to HIV viral particles or not.

Having a control group comprising HEU children who were not on CTX prophylaxis would have allowed us to clearly specify if what was observed in the HEU on CTX prophylaxis was indeed an effect of CTX on the proportion of various cell subsets and not due to *in utero* HIV exposure. However, ethically it was not possible to have such a group since the current Malawi Government guidelines clearly state that every HEU child should be on CTX for the first year of life for the obvious beneficial effects. We therefore used HUU recruited from same communities as controls instead. 

## Conclusions

In conclusion, we have shown that there are no long term alterations in the proportion of B and T cellsubsets in HEU children compared to HUU children suggesting that HEU children have a robust cellmediatedimmune system during the first year of life.

## Abbreviations

ART: Antiretroviral therapy
CTX: Cotrimoxazole
HEU: HIV-exposed but uninfected
HUU: HIV-unexposed and uninfected
PMTCT: Prevention of mother to child transmission
